# A Smartphone App to Promote Healthy Weight Gain, Diet, and Physical Activity During Pregnancy (HealthyMoms): Protocol for a Randomized Controlled Trial

**DOI:** 10.2196/13011

**Published:** 2019-03-01

**Authors:** Pontus Henriksson, Johanna Sandborg, Marie Blomberg, Christina Alexandrou, Ralph Maddison, Kristin Silfvernagel, Hanna Henriksson, Marja H Leppänen, Jairo H Migueles, Linnea Widman, Kristin Thomas, Ylva Trolle Lagerros, Marie Löf

**Affiliations:** 1 Department of Biosciences and Nutrition Karolinska Institutet Huddinge Sweden; 2 Department of Medical and Health Sciences Linköping University Linköping Sweden; 3 Department of Obstetrics and Gynecology Linköping University Linköping Sweden; 4 Department of Clinical and Experimental Medicine Linköping University Linköping Sweden; 5 Institute for Physical Activity and Nutrition Deakin University Burwood Australia; 6 Division of Psychology Department of Behavioural Sciences and Learning Linköping University Linköping Sweden; 7 Faculty of Sport and Health Sciences University of Jyvaskyla Jyvaskyla Finland; 8 Department of Physical and Sports Education Faculty of Sport Sciences University of Granada Granada Spain; 9 Department of Biostatistics Institute of Environmental Medicine Karolinska Institutet Stockholm Sweden; 10 Clinical Epidemiology Unit Department of Medicine Karolinska Institutet Solna Sweden; 11 Obesity Center Academic Specialist Center Stockholm Health Services Stockholm Sweden

**Keywords:** telemedicine, pregnancy, gestational weight gain, diet, exercise, smartphone, mobile phone

## Abstract

**Background:**

Excessive gestational weight gain is common and associated with adverse outcomes both in the short and long term. Although traditional lifestyle-based interventions have shown to mitigate excess gestational weight gain, little is known about whether mobile Health (mHealth) apps can promote healthy weight gain, diet, and physical activity during pregnancy.

**Objective:**

The primary aim of the HealthyMoms trial is to determine the effectiveness of a smartphone app (HealthyMoms) for mitigating excess gestational weight gain during pregnancy. Secondary aims are to determine the effectiveness of the app on dietary habits, physical activity, body fatness, and glycemia during pregnancy.

**Methods:**

HealthyMoms is a two-arm randomized controlled trial. Women are being recruited at routine visits at the maternity clinics in Linköping, Norrköping and Motala, Sweden. Women are randomized to the control or intervention group (n=150 per group). All women will receive standard care, and women in the intervention group will also receive the HealthyMoms smartphone app.

**Results:**

Recruitment of participants to the trial was initiated in October 2017, and 190 women have so far completed the baseline measurement. The baseline measures are estimated to be finalized in December 2019, and the follow-up measures are estimated to be completed in June 2020.

**Conclusions:**

This project will evaluate a novel smartphone app intervention integrated with existing maternity health care. If successful, it has great potential to be implemented nationally in order to promote healthy weight gain and health behaviors during pregnancy.

**International Registered Report Identifier (IRRID):**

DERR1-10.2196/13011

## Introduction

### Background

Studies from developed countries including Sweden report that almost half of all pregnant women exceed the Institute of Medicine’s recommendations for gestational weight gain [1-3]. This is concerning because excess gestational weight gain is associated with negative outcomes for maternal and child health both in the short term (eg, gestational diabetes, preeclampsia, large for gestational age infants, and cesarean delivery) and long term (postpartum weight retention and obesity in the offspring) [1,2,4-6]. Thus, prevention of excess gestational weight gain is a public health priority. A 2015 Cochrane review concluded that diet, exercise, or both interventions reduced the risk of excess gestational weight gain by 20% [7]. These results are promising and suggest that lifestyle-based interventions can mitigate excess gestational weight gain during pregnancy. Notwithstanding these positive findings, previous studies have used traditional face-to-face education by means of individual or group counselling with heavy reliance on intensive support from clinical providers, which limits the scalability of these programs [7]. Considering the potential public health benefits, it is important to develop interventions that can reach a large proportion of the target group.

In the past 5-10 years, there has been a large increase in research on the use of mobile phones (including smartphones) for delivering behavior change interventions. The benefits of such mobile health (mHealth) programs are that they can be delivered anywhere at any time, they are interactive, and they can be tailored to meet people’s needs. Several reviews have concluded that mHealth programs may be effective for achieving changes in behavior and weight loss [8,9]; mHealth interventions may therefore also be effective in promoting a healthy lifestyle and gestational weight gain in pregnant women [10]. Additionally, mHealth offers a potential solution to provide support for pregnant women, in general, since mobile phones are commonly accessible, irrespective of socioeconomic status [10]. However, to date, mHealth interventions to promote healthy gestational weight gain in healthy pregnant women are scarce [11]. Therefore, further studies are warranted to determine the utility of mHealth for mitigating gestational weight gain during pregnancy.

### Aim

This paper reports the study design and methods of the HealthyMoms trial (trial registration: ClinicalTrials.gov NCT03298555). The primary aim of the HealthyMoms trial is to determine the effectiveness of a smartphone app (HealthyMoms) for mitigating excess gestational weight gain during pregnancy. Secondary aims are to determine the effectiveness of the app on dietary habits, physical activity, body fatness, and glycemia during pregnancy.

## Methods

### Study Design

**Figure 1:**
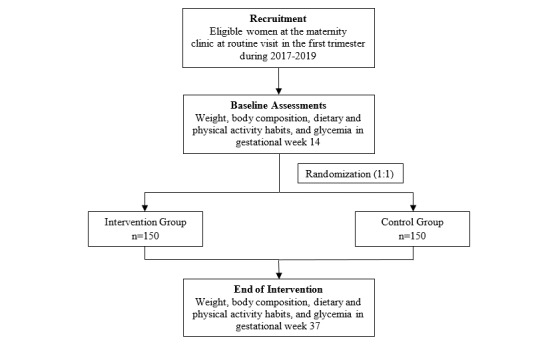
Study design of the HealthyMoms trial.

The HealthyMoms trial is a two-arm parallel randomized controlled trial. The intervention group will receive standard care plus the HealthyMoms app (a 6-month program delivered through their smartphones). Participants in the control group will receive standard antenatal care provided by the maternity health care system. The study will be conducted and described according to the Standard Protocol Items: Recommendations for Interventional Trials 2013 statement [12] and the CONSORT-EHEALTH checklist [13]. The outline of the HealthyMoms trial is presented in Figure 1. The baseline measurements and randomization are conducted in gestational week 14, and the intervention will be initiated in gestational week 15. Measurements after the intervention will be conducted in gestational week 37.

### Eligibility and Recruitment

Eligible pregnant women are identified at the routine visits in the first trimester at the maternity clinics in Linköping, Norrköping, and Motala in Sweden. Inclusion criteria are a single pregnancy, age of 18 years or above, and ability to speak and read Swedish sufficiently well in order to understand the content of the HealthyMoms app and provide informed consent. Exclusion criteria are previously diagnosed eating disorder, pre-pregnancy diabetes, other medical conditions, or pharmacological treatments prior to pregnancy that may affect body weight.

### Randomization and Blinding

Following baseline measurement, participants will be randomly allocated to either the intervention or control group in a 1:1 ratio using restricted randomization (with a block size of 2) generated using STATA (version 13; StataCorp, College Station, TX) by a statistician. Opaque envelopes will be used for allocating participants to the respective groups, ensuring allocation concealment. Due to the nature of the intervention, participants will not be blinded to their allocation.

### Intervention

#### Overall

**Figure 2:**
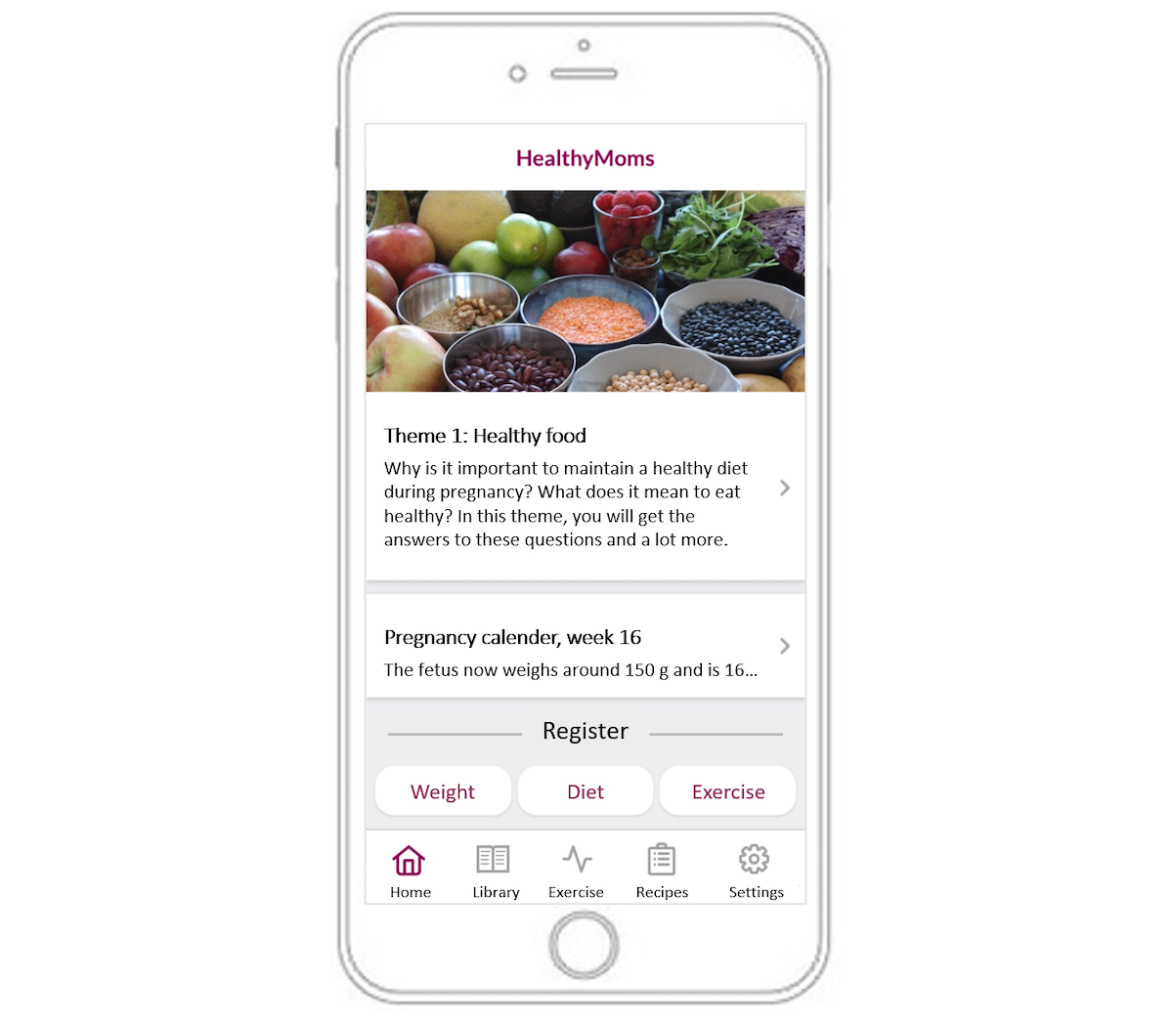
Screenshot of the HealthyMoms app.

The HealthyMoms app is a comprehensive 6-month program aimed at mitigating excess gestational weight gain by promoting a healthy diet and physical activity. A screenshot of the HealthyMoms app is shown in Figure 2. The app is Android and IOS compatible. Participants who are allocated to the intervention group will receive a text message with a link to a website, which they can access through their phone. Using the website, participants will be instructed to register and download the app from Google Play or App Store.

#### Development

The HealthyMoms app utilizes the same platform (ScientificMed Tech AB) [14] and structure as our previously validated app targeting parents with preschool aged children [15]. The specific content and features for the HealthyMoms app were developed by a multidisciplinary team with expertise in nutrition, behavioral science, obstetrics, psychology, physiotherapy, and physical activity. Intervention development was an iterative process, whereby input from target group members and experts as well as behavior change theory (social cognitive theory [16] and behavior change techniques [17]) were considered. Specifically, input on the content and features were discussed in semistructured interviews with pregnant women and women who had recently given birth (n=10). Further, content regarding maternal and fetal development as well as dietary recommendations were reviewed by midwives and relevant experts at the National Food Agency, Sweden. Theory was used to inform the development of the intervention. For example, key behavior change techniques such as shaping knowledge (eg, general information on healthy diet, physical activity, and gestational weight gain), goals and planning (eg, goal setting and identification of barriers), and feedback and monitoring (eg, self-monitoring of behavior and feedback on behavior).

#### Content and Use

The HealthyMoms app delivers a comprehensive program of information and push notifications based on evidence-based recommendations for a healthy diet [18], physical activity [19], and weight gain [1] in pregnant women. The app consists of seven features: weekly themes; push notifications; self-monitoring and feedback features of gestational weight gain, diet, and physical activity; recipe feature; exercise feature; pregnancy calendar; and app library (eg, frequently asked questions and practical tips).

The program addresses 12 *themes* that change every other week: healthy foods, healthy weight gain during pregnancy, physical activity and exercise during pregnancy, how to change a habit, cravings, fruits and vegetables, nutrition for the mother and baby, the third trimester, the reasons why we eat, exercise at the end of pregnancy, how to keep a new habit, and the time after the delivery. Participants are alerted when a new theme is initiated via a *push notification* every other week. In total, participants receive eight push notifications for every theme (one message every other day and an additional message). These messages consist of, for example, factual information or behavior change guidance, support and guidance on healthy habits (eg, diet and physical activity), weight gain during pregnancy, strategies for behavior change, encouragement, key messages, and reminders.

The app includes a *self-monitoring and feedback feature*, whereby diet, physical activity, and weight gain are reported by the user, which is followed by tailored feedback in the form of graphical illustration and text. Self-monitoring of diet involves answering five questions each week on the intake of fruits, vegetables, sweets, and sugary drinks. Participants receive feedback presented in a graph and in text with a “traffic light” (green: reached the recommendation, yellow: almost reached the recommendation, red: far from reaching the recommendation). Self-monitoring of physical activity entails setting a physical activity goal (in activity minutes) and reporting of weekly activity levels. Individual self-monitoring data are summarized graphically, and participants can see if they are close to or if they have met their own goal as well as the recommendation for physical activity (150 minutes/week) each week [19]. The participants also receive feedback in text format and with the “traffic light,” as described above. Self-monitoring of weight consists of reporting current weight. The weight gain is presented graphically; from gestational week 22 until the end of pregnancy, a green field is visible, showing the participant’s weight gain in relation to their individual recommended gestational weight gain (in accordance with the Institute of Medicine recommendation and calculated from their pre-pregnancy body mass index).

The app also includes an *exercise feature* and *a recipe feature*. The exercise feature includes information, tips, and exercise programs for the different stages of pregnancy in both text and video format. The recipe feature has a library of healthy recipes and weekly menus. The HealthyMoms app also includes a *pregnancy calendar* that is updated every week. It consists of information on fetal and maternal development during pregnancy as well as texts to the partner (eg practical information, encouraging, and supportive texts). Lastly, the app includes a *library* of practical tips, frequently asked questions, and useful links.

### Sample Size and Power Considerations

Power for an independent *t* test was calculated *a priori* using G*Power 3.1 [20]. A total of 226 women (113 in each group) completing the measurements after the intervention provides 80% power (α=.05, two-sided), assuming a common SD in gestational weight gain of 4 kg [21] to detect a difference of 1.5 kg between the groups. Given our previous experiences [22], we anticipate that no more than 25% of the women will drop out of the study or have a child prematurely. Thus, we will recruit 300 women (150 in each group) to participate in the study.

### Primary outcome

#### Gestational Weight Gain

The primary outcome gestational weight gain is calculated as the measured body weight in gestational week 37 minus the measured body weight in gestational week 14. Body weight is recorded using standardized procedures when the participant is wearing only underwear. We will also analyze gestational weight gain as within or above the Institute of Medicine-recommended levels by using the recommended weekly gains in body weight during the second and third trimester [1].

### Secondary Outcomes

#### Dietary Intake

Dietary intake is measured using Riksmaten FLEX [23], adapted to pregnant women. It combines three 24-hour recalls and a food-frequency questionnaire to measure intakes of foods and drinks. Intakes of energy, macronutrients, and micronutrients are calculated through linkage with the amount of foods assessed through Riksmaten FLEX to the food composition database of the National Food Agency of Sweden [24].

#### Physical Activity and Sedentary Behavior

Physical activity and sedentary behavior are measured objectively over seven consecutive 24-hour periods with a wrist-worn triaxial accelerometer, Actigraph wGT3x-BT (ActiGraph, Pensacola, FL). Wrist-worn accelerometers including the ActiGraph [25] have been validated previously in adults [25,26] and pregnant women [27]. The recorded movements will be processed as previously described [27,28] and converted to time spent on different activity levels (ie, sedentary behavior as well as light, moderate, and vigorous physical activity) using appropriate cutoffs.

#### Body Fatness

Body fatness is measured using Bod Pod (COSMED, Rome, Italy), as previously described [29]. This method utilizes air-displacement plethysmography to measure body volume. Body density is then calculated using the measured body volume and body weight. Subsequently, body fatness is calculated using the gestational-age specific densities for fat-free mass published by Van Raij et al [30]. These values from fat-free mass density have previously been shown to be suitable for use throughout pregnancy [31-33].

#### Glycemia, Gestational Diabetes, and Insulin Resistance

A blood sample is drawn after an overnight fast, and glucose, insulin, and lipid profile (triglycerides and high-density and low-density lipoprotein cholesterol) will be analyzed at the Department of Clinical Chemistry, Linköping University Hospital. Furthermore, systolic and diastolic blood pressure will be assessed in a resting state, following standardized procedures. Gestational diabetes is defined according to the International Association of Diabetes and Pregnancy Study Group’s cutoff [34]. Furthermore, insulin resistance, as assessed by the HOMA-IR (homeostasis model assessment - insulin resistance) will be calculated according to the study by Matthews et al [35].

### Other Measurements

#### Demographic Information

The women and their partners complete separate demographic questionnaires, which include information regarding age, country of birth, occupation, educational attainment, previous pregnancies, smoking habits, dietary and physical activity habits, overall health, and use of medications.

#### Physical Fitness and Subjective Physical Activity

Cardiorespiratory fitness is assessed using the 6-minute walk test, which measures the maximum distance (in meters) the woman can walk in 6 minutes back and forth in a 30-m corridor [36]. Participants are instructed to walk as fast as possible during the test, but they are also informed that they may slow down or rest, if necessary. The 6-minute walk test is only conducted at the baseline measurement, whereas all other measures of physical activity and fitness will be conducted both at the baseline measurement and at gestational week 37. Upper body muscular strength is measured using the handgrip strength test, where the woman is asked to squeeze a dynamometer (TKK 5001; Grip-A, Takei, Tokyo, Japan) as hard as she can with her hand for a few seconds [37]. Physical fitness is also assessed subjectively using an adapted version of the international fitness scale, which includes cardiorespiratory fitness, muscular strength, speed and agility, flexibility, and overall fitness using 5-point Likert-scale questions [38]. As a complement to the objective physical activity data, we also collect data of subjective physical activity using two questionnaires: the short-form International Physical Activity Questionnaire, where participants recall their physical activity over the past 7 days [39], and a 7-day modified version of a questionnaire used by Bexelius et al [40].

#### Maternal and Infant Body Fatness Postpartum

At 1-2 weeks postpartum, maternal body fatness is measured using air-displacement plethysmography (as described above) utilizing suitable postpartum fat-free mass density values [32]. At the same time, infant body composition is measured using air-displacement plethysmography (Pea Pod, COSMED), as described in detail previously [22]. Briefly, body weight and body volume are measured by the Pea Pod. Body fatness is then calculated using the measured body density and appropriate fat-free mass hydration factors [41].

#### App Usability and Process Evaluation

Participants in the intervention group will complete a questionnaire including questions on their use and perception of the app with regard to usability, design, features, and overall satisfaction. Furthermore, semistructured interviews (audio) will be conducted within a subsample (n=10-20) of women in the intervention group to gain additional insight about their perceptions of the HealthyMoms app. The interviews will be transcribed and analyzed using thematic analysis, as described elsewhere [42].

### Statistical Analyses

Imputed data will be continuously checked against source data, and range checks will be implemented. The primary outcome will be gestational weight gain (kilogram). Analyses will be conducted according to the principles of intention-to-treat and per-protocol procedures using independent *t* tests to test whether gestational weight gain is statistically different between the groups. In the intention-to-treat analysis, missing data will be imputed using multiple imputations with chained equations [43]. In the per protocol, patients will be defined as completers if they have gestational weight gain data (body weight measured at baseline and gestational week 37) and if they have downloaded and used the HealthyMoms app at least once. Furthermore, linear regression will be considered to adjust estimates for the pre-pregnancy body mass index group (ie, underweight and normal weight vs overweight and obese), socioeconomic status (university degree vs no university degree), and parity (0 vs ≥1). In this analysis, treatment will be used as a categorical variable, with the control group as the reference. This model will also be extended to include interactions (one at a time) of the treatment with the pre-pregnancy body mass index, socioeconomic status, and parity, since we want to investigate whether the effect of the intervention differs depending on these factors. In a sensitivity analysis, we will also exclude women diagnosed with gestational diabetes and pre-eclampsia before the follow-up measurement, since these women may have received intensive diet/physical activity support or medication or may have edema, all of which may influence the outcome variables (eg, body weight, diet, physical activity, and glycemia).

### Ethical Approval

The HealthyMoms trial was approved by the Regional Ethical Review Board in Linköping, Sweden on April 24, 2017 (DNR: 2017/112-31), with an amendment on May 4, 2018 (DNR: 2018/262-32). All women will provide written informed consent before entering the study. The mother and father/partner will provide informed written consent before any measurements of their infant are performed.

## Results

The HealthyMoms app was finalized in September 2017. Recruitment of participants to the trial was initiated in October 2017, and 190 women have so far completed the baseline measurement. The baseline measurements are estimated to be finalized in December 2019, whereas the follow-up of the mothers and infants is estimated to be completed in June 2020.

## Discussion

The HealthyMoms trial will examine whether a novel mHealth app can mitigate excess gestational weight gain and promote healthy dietary habits, physical activity, healthy levels of body fatness, and normal glycemia. The trial has several strengths including the objective and accurate methodology to measure physical activity and body composition, close collaboration with existing maternity health care services, and a large sample size. The latter represents a distinct strength, considering that previous studies that have investigated the effect of a mHealth app on gestational weight gain have been relatively small pilot studies, although some have shown promising results. For instance, in a study of 100 overweight and obese Australian women, Wilcox et al [44] found that women in the intervention group had significantly lower gestational weight gain than women in the control group. Similarly, in a trial of 54 overweight/obese American women, Redman et al [45] found lower gestational weight gain in those receiving an intensive intervention delivered in-person or via smartphone as compared to those receiving usual care [45]. However, Chao et al [46] did not find any effect of a telemedicine intervention on gestational weight gain in 41 American women with overweight or obesity. Hence, the HealthyMoms trial will make an important contribution to the existing literature, considering the study size and that normal weight women are included in the study. This is important because excessive gestational weight gain is common among women having normal weight before pregnancy [1,3]. Finally, the intervention content is grounded in the social cognitive theory [16] and uses well-recognized behavior change techniques [17].

The HealthyMoms trial also has several limitations. First, although all eligible women are approached to enter the study, it is possible that an overrepresentation of highly educated, health conscious, and normal-weight women enter the trial. However, we will also examine whether there are any differences in the effect of the intervention in terms of maternal pre-pregnancy body mass index, educational attainment, and parity. Second, due to the time and budget constraints, we did not individually tailor push notifications; however, women have an individually tailored weight gain chart depending on their pre-pregnancy body mass index. Third, due to the thorough baseline measurement and to ensure that most women would be able to participate in the trial, women will receive the HealthyMoms app in gestational week 15. However, if the app is proven effective, an earlier introduction in pregnancy should be considered when fully implementing the app as a supportive tool in maternity health care. Finally, women need to be able to speak and read Swedish sufficiently well to understand the content of the HealthyMoms app in order to be eligible for participation. Hence, we aim to translate and modify the HealthyMoms app in order to make it widely accessible.

Excessive gestational weight gain is a major public health issue globally [1,2] and in Sweden [3], and it is associated with adverse outcomes both during pregnancy as well as later in life [1,2,4-6]. If effective, the HealthyMoms app has potential to be implemented in maternity clinics nationally and offer an evidence-based intervention program to women at relatively low costs. This is of particular importance, given the ubiquity of mobile phone ownership, irrespective of socioeconomic status [10].

## Appendix

Multimedia Appendix 1 Peer-reviewer report 1.

Multimedia Appendix 2 Peer-reviewer report 2.

## Abbreviations

HOMA-IR: homeostasis model assessment - insulin resistance
mHealth: mobile health
