# Supplementary material for: A Smartphone App to Promote Healthy Weight Gain, Diet, and Physical Activity During Pregnancy (HealthyMoms): Protocol for a Randomized Controlled Trial
Source: JMIR Res Protoc. 2019 Mar 1;8(3):e13011. doi: 10.2196/13011 (PMC6418485; doi:10.2196/13011)
Supplement: Multimedia Appendix 1 [file resprot_v8i3e13011_app1.pdf]

Beredningsgruppens yttrande

2017-00088	Pontus Henriksson	Beredningsgrupp: HBPost17

**Utlysningsnamn:** Postdoc 2017	**Bidragsform:** Postdoc
**Projekttitel (svenska):** Healthy Moms: främjande av hälsosam viktökning och livsstil under graviditet	**Sökt inriktning:** Fritt

Vetenskaplig kvalitet på avhandlingen och eventuella andra vetenskapliga arbeten

The dissertation for ph.d. is of high quality, good methods and the papers are published in well-known, mostly high-ranked peer-reviewed scientific journals. The candidate is first author of seven papers and has contributed to another five with research questions of high originality.

Den sökandes kompetens

The candidate is nutritionist with a broad expertise in physiological measurements. He spent 15-16 months on a post doctor stay in Spain where he gained knowledge in designing RCT's. He has collaborated with excellent research teams and demonstrates skilled insight in the topic of the research project. The project is divided in two parts, the second one is a registry study. It is not, clear however, whether he has any experience with managing such datasets and the necessary skills in registry based research and epidemiology that will be needed.

Projektets samhällsrelevans

The application involves two parts, one RCT and one registry-based. The main research topic is to study gestational weight gain, whether excessive (for the majority) or insufficient. The research questions are of high societal relevance and is expected to contribute with needed knowledge that could be applicable in the primary health care and pregnancy ward. Whether application of mHealth in preventive medicine and care is suitable and a simple (and cheap) measure to help the individual pregnant woman take health promoting choices is of high societal relevance. One specific aim to study gestational weight variations among migrant women in Sweden, both in the RCT and in the registry study.

Genus och mångfald

The intervention and pregnant Swedish and migrant women, but the project also aims at study gender differences among the infants. The project is partly aiming specifically at migrant women and has the potential to contribute to improved health equity among different ethnic groups in Sweden.

Planer för kommunikation och samverkan

Plans for dissemination are well described. A part from scientific publications there are plans for popular communications. The research team has a broad network and the individual researchers are member of national expert groups and scientific associations where the research and its results also will be spread.

Sammanfattande bedömning av projektets genomförbarhet och värde

The project seems well feasible, the candidate will take part in a well established research team on an already funded intervention involving a smartphone app. In this part of the study he will be supervisor of a ph.d.candidate. Additionally, he will perform a registry study in the Swedish Pregnancy Registry in order to study the prevalence of unhealthy gestational weight gain according to country of birth and socio-economic status. The description of the project and the candidate's task is well-written, power calculations of the RCT indicate sufficient power to detect differences between the group at statistical significant levels. One small question mark whether the candidate has the needed skills in registry studies, but this will presumably be taken care of within the experienced team. The project has high societal relevance and is recommended for funding if the budget allows it.

Förslag (bevilja, bevilja i mån av medel, avslå)

Approve subject to funding
