# Supplementary material for: A Smartphone App to Promote Healthy Weight Gain, Diet, and Physical Activity During Pregnancy (HealthyMoms): Protocol for a Randomized Controlled Trial
Source: JMIR Res Protoc. 2019 Mar 1;8(3):e13011. doi: 10.2196/13011 (PMC6418485; doi:10.2196/13011)
Supplement: Multimedia Appendix 2 [file resprot_v8i3e13011_app2.pdf]

Beredningsgruppens yttrande

2016-01147	Marie Löf	Beredningsgrupp: MH-G2

Utlysningsnamn: Forskningsbidrag Stora utlysningen 2016 (Medicin och hälsa)

Projekttitel (svenska): HealthyMoms -en mobiltelefonsapplikation för att främja hälsosam viktsökning och goda kost-och aktivitetsvanor under graviditet samt motverka fetma hos mammor och barn: en randomiserad studie

Bidragsform: Projektbidrag

Sökt inriktning: Fri

Novelty and originality 5

1 - Poor, 2 - Weak, 3 - Good, 4 - Very Good, 5 - Very good to excellent, 6 - Excellent, 7 - Outstanding

The project will develop and use novel technology (smartphones) to evaluate if an app could have an impact on change of behaviour regarding excessive weight gain through adapting physical activity and food intake during pregnancy (last 6 months). It is expected to create promising novelty regarding a well known public health problem.

Scientific quality of the proposed research 5

1 - Poor, 2 - Weak, 3 - Good, 4 - Very Good, 5 - Very good to excellent, 6 - Excellent, 7 - Outstanding

The applicant is addressing an important public health problem. A clear background is presented, although the description could be more convincing that the project will reach the group that have the highest risk for long term morbidity of obesity (women of low SES, migrants). General design, including time schedule, seems to be optimal for implementing the proposed project.

Merits of the applicant(s) 5

1 - Poor, 2 - Weak, 3 - Good, 4 - Very Good, 5 - Very good to excellent, 6 - Excellent, 7 - Outstanding

The applicant has an earlier experience of this type of intervention studies. A well qualified team includes also international collaboration, although roles of co-investigators in the project not fully specified. The applicant has an extensive publication record, with numerous first or senior author positions. Successful external grants (e.g. from VR, Forte).

Feasibility 3

1 - Not feasible, 2 - Partly feasible, 3 - Feasible

Project appears generally feasible.

Scientific report 5

1 - Poor, 2 - Weak, 3 - Good, 4 - Very Good, 5 - Very good to excellent, 6 - Excellent, 7 - Outstanding

Earlier findings are presented and they resulted in several publications.

Overall assessment of the scientific quality 5

1 - Poor, 2 - Weak, 3 - Good, 4 - Very good, 5 - Very good to excellent, 6 - Excellent, 7 - Outstanding

Original and important translational research project, adapting existing technologies towards an in-need population. It can have implications in a future medical practice.
